# Insight Into Microbial Community Aerosols Associated With Electronic Waste Handling Facilities by Culture-Dependent and Culture-Independent Methods

**DOI:** 10.3389/fpubh.2021.657784

**Published:** 2021-04-06

**Authors:** Yimin Pan, Qiaoqiao Ren, Pei Chen, Jiguo Wu, Zhendong Wu, Guoxia Zhang

**Affiliations:** ^1^Guangdong Provincial Key Laboratory of Tropical Disease Research, School of Public Health, Southern Medical University, Guangzhou, China; ^2^Guangdong-Hong Kong-Macao Joint Laboratory for Contaminants Exposure and Health, Guangzhou, China

**Keywords:** e-waste dismantling site, waste transfer station, bioaerosol, bacterial diversity, culture

## Abstract

Airborne microorganisms in the waste associated environments are more active and complex compared to other places. However, the diversity and structure of airborne bacteria in waste-associated environments are still not clearly understood. The purpose of this study was to assess airborne bacterial community in electronic waste dismantling site and a waste transfer station based on culture-dependent and culture-independent methods. A total of 229 isolates were obtained from four airborne sites collected from residential area, electronic industrial park, and office area in or near an electronic waste dismantling site and a waste transfer station in Southern China in the morning, afternoon, and evening. Most of the isolates were isolated from air for the first time and 14 potentially novel species were identified by Sanger sequencing. Bacterial communities in waste-associated bioaerosols were predominated by Proteobacteria and Bacteroidetes. Abundant genera (>1%) included Paracaedibacteraceae (uncultured EF667926), *Ralstonia, Chroococcidiopsis*, Chitinophagaceae (uncultured FN428761), *Sphingobium*, and *Heliimonas*. One-third of the species in these genera were uncultured approximately. Differences community structure existed in airborne bacterial diversity among different sampling sites. These results showed that waste-associated environments have unique bacterial diversity. Further studies on such environments could provide new insights into bacterial community.

## Introduction

Bacterial diversity is central to explore the relationship between bacterial aerosols and human health or public health, mainly reflecting in both direct and indirect aspects (1). Bacterial diversity directly affects human health, which the rich bacterial community can prevent from certain metabolic diseases, gastrointestinal diseases or skin mucous membrane diseases (2, 3). Bacterial diversity indirectly alters public health. The isolation of microbial strains from bacterial communities is an essential precondition for the development of microbial function, exhibiting vast functional diversity. New unique functions are continuously discovered as the number of known species increases (4). Studying new communities can therefore reveal previously unknown functions including exploitation of novel compounds, potential cause of drug side effects, therapeutic target, and the source of inflammatory disease (5–7). Studies on bacterial diversity can therefore be expected to provide important new insights with applications in biotechnology, human health, and public health (7).

The structure and diversity studies on bacterial bioaerosols focus on different environments, such as landfills, composting facilities, sewage treatment plants, farms, or hospitals, bio-waste and recycling industry (8–11). Electronic waste (e-waste), i.e., waste derived from electrical or electronic equipment, is chemically and physically distinct from other municipal waste (12). At present, microbial diversity in electronic waste areas has been extensively studied, but most of them focus on soil and water environments. Waste transfer stations are mainly used for temporary storage of municipal waste, and the air bacteria around them may differ from those associated with e-waste. Previous studies provide the concentration and distribution of noxious gas in waste transfer station (13). However, with very few researches on e-waste bioaerosols and waste transfer stations, the diversity and structure of airborne bacteria in waste handling facilities-associated environments, such as, electronic waste and waste transfer station, are still not clearly understood.

Aerosols are also often rich in unidentified microorganisms and over 90% of airborne microorganisms were uncultivable (14). Molecular tools are widely used in airborne bacteriological research and many uncultivable bacteria have recently been discovered using next-generation sequencing technology (15). However, culture-dependent methods remain vital in bacteriological research because isolating and cultivating pure strains provides insights into their phenotype, metabolism, and genetic functions, potentially revealing new applications in biotechnology, human healthcare, and industry (7, 16–18). Greater diversity could potentially be uncovered by combining culture and non-culture methods (19). However, there have been very few studies on bacterial diversity in airborne microorganisms using such a combined approach.

In the present study, we hypothesized that the waste-associated environments have particular bacterial diversity. To test this hypothesis, aerosol samples were collected from an e-waste dismantling site in South China and a waste transfer station near the Southern Medical University in Guangzhou. The collected bacteria were isolated and identified by culture method, and non-culture method was used for the detection of the bacterial communities on structure and diversity. The results obtained provide new insights into bacterial diversity in e-waste-related aerosols.

## Materials and Methods

### Sample Collection

Sampling was mainly conducted at an e-waste dismantling site in South China (23.32°N, 116.36°E) and a waste transfer station near the Southern Medical University in Guangzhou (23.18°N, 113.33°E) in November 2019. Four different functional areas were chosen to study the biodiversity of bacterial aerosols in waste-related environments. The sampling sites included a residential area (A), an electronic industrial park (B), an office area (C), and a waste transfer station (D) (Supplementary Figure 1). Sampling at the points D was performed in the morning (Dm) and afternoon (Da), and at night (Dn). Sampling at points A, B, and C was performed in the morning, noon, and evening, respectively, on a different day with similar meteorological conditions as point D. The residential area, electronic industrial park (e-industrial park), and office area were in the town of Guiyu, which is an e-waste site. These three functional areas were affected by e-waste to varying degrees but were all regarded as e-waste disassembly sites in this study. Waste stored at the waste transfer station included kitchen waste, plastic, paper, metal products, construction waste, and so on from surrounding schools, communities, and hospitals.

Air sampling was carried out simultaneously using two different samplers at each sampling site and was performed in triplicate at each site. The sampler was 2–3 m from the waste and 1.5 m above the ground. Culturable airborne bacteria were pumped using six-stage Andersen Cascade Impactors at a flow rate of 28.3 L/min for 10 min. The Andersen Impactor was equipped with nutritional agar plates and stage 1–6 filters with cut-off diameters of 7.0, 4.7, 3.3, 2.1, 1.1, and 0.65 μm, respectively. Non-culturable bacteria were collected using a high-flow portable bioaerosol aerosol sampler (Beijing Dingblue Technology Co., Ltd) with a 2 μm cutoff size at a flow rate of 1,000 L/min for 30 min. The high-flow sampler used not nutritional agar but a plate wrapped in aluminum foil with 800 μL of mineral oil. The detailed information of the high-flow portable bioaerosol aerosol sampler can be referred to Li et al. (20). In total, six samples of airborne bacteria (both culturable airborne bacteria and non-culturable bacteria) were collected. Concentrations of PM_2.5_ and PM_10_ (particles with aerodynamic diameters below 2.5 and 10 μm, respectively) and meteorological parameters were acquired from the Guangdong Meteorological Service. Detailed environmental data on all the sampling sites is presented in Supplementary Table 1 in the Supplementary Material.

### Isolation of Culturable Bacteria

Colonies that grew on a nutrient agar plate were incubated at 37°C for 24 h. Single colonies were obtained by repeated plate streaking. All purified colonies were identified by Sanger DNA sequencing performed by the GENEWIZ Company. The 16S rRNA gene sequences were deposited in GenBank under accession Nos. MT214102–MT214329, MT218357, and MT214107. Sanger sequencing results were compared to the NCBI database to test the 16S rRNA gene sequence similarity. Sequences with similarity below 98.65% were sent to the MAGIGENE company for Whole genome sequencing. Calculation of Average Nucleotide Identity and DNA-DNA hybridization (<95 or 70%, respectively) from whole genome sequencing data to identify potentially novel bacteria (These data were given in another article).

### Sequencing of Non-culturable Bacteria

Genomic DNA from aerosol samples was extracted using the phenol-chloroform method (21). The 16S rRNA gene V4 region was amplified by the following barcoded primers (shown from 5′ to 3′): V4F, GTGYCAGCYMGCCYGCGGYTAA and V4R, GGACYTACNYVGGGYTWTCYTAAT. The following PCR conditions were used: initial denaturation at 94°C for 5 min followed by 30 cycles of denaturation at 94°C for 30 s, annealing at 52°C for 30 s, extension at 72°C for 45 s and a final extension at 72°C for 5 min. All PCR products were sequenced on an Illumina Hiseq 2,500 using 500 cycle version 2 reagent kits (The Beijing Genomics Institute) at Zhujiang Hospital (Guangzhou, China). The method of sample detecting, library construction, end repair, A-tailing, adaptor ligation, size-selected library, and products purification, validation of the library, sequencing libraries Amplification, and data processing were performed as previously described (22). For bacteria that could not be classified, their genetic sequences were searched against the NCBI and EZbiocloud database to identify them at the genus level. Three replicate samples were collected at each site, and each sample was sequenced five times. The complete DNA sequence dataset was deposited in the Sequence Read Archive (SRA) under study accession no. PRJEB38065.

### Statistical Analysis

GraphPad Prism (version 8.4.3) was used to plot box plots, bar charts, and heatmaps related to alpha diversity. Principal coordinate analysis plots, Cladogram, and Venn diagram related to beta diversity were performed in R (version 3.2.2) using the “capscale” function in the “vegan” R package. The SPSS software (version 25) was used for data analysis. The bacterial abundance among different sampling sites was assessed by one-way analysis of variance. Pearson Correlation analysis was conducted to explore the correlations between culturable airborne bacterial concentration and weather parameters. For those data that do not conform to the normal distribution, such as, alpha diversity indices, the Kruskal-Wallis H test was performed. Beta diversity was then analyzed with a permutational multivariate ANOVA (PERMANOVA). The statistical significance threshold was set at *P* < 0.05.

## Results

### Concentrations and Size Distributions of Airborne Bacteria

The concentrations and size distributions of culturable airborne bacteria at sampling sites A, B, C, Dm, Da, and Dn are presented in Supplementary Table 1 and Supplementary Figure 2. Bacteria concentrations were highest in the office area (744 ± 78 CFU/m^3^) and lowest in the e-industrial park (70 ± 5 CFU/m^3^). The particle size distributions at sites A, B, and C were similar, peaking in stages 5 (1.1–2.1 μm) or 6 (0.65–1.1 μm). The average daily concentration of bacterial aerosols in the waste transfer station was 463 ± 316 CFU/m^3^ (m ± sd). There was pronounced intraday variation in bacterial concentration and particle size, with the highest concentration occurring in the afternoon and the lowest in the morning. For the waste transfer station, 80% of the bacteria were deposited in stages 4 and 5 in the morning. The dominant particle size increased over the course of the day, from small to medium or even large; by nightfall, the number of bacteria >7 μm in diameter exceeded 55%.

### 16S rRNA Gene Identification of Isolates

**Figure 1:**
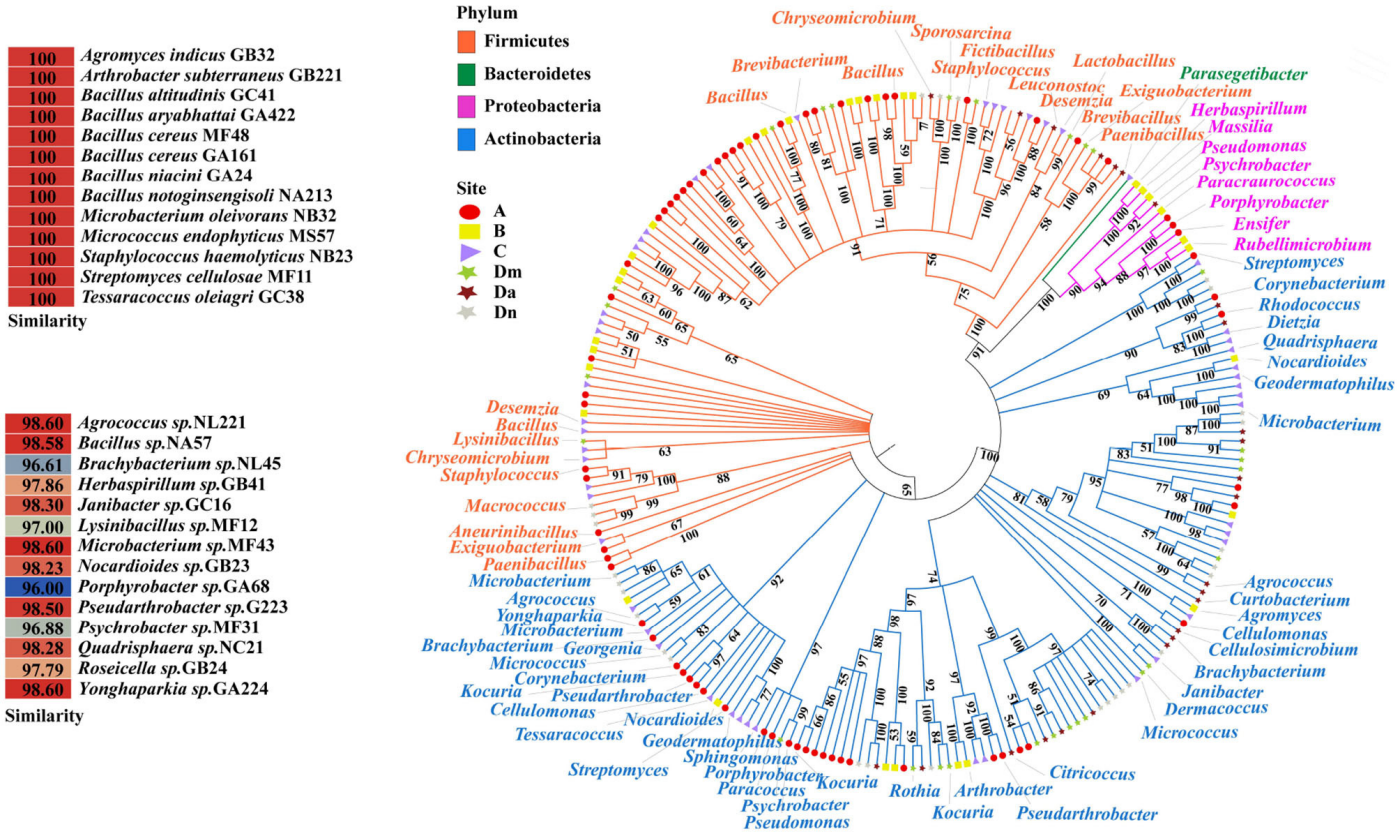
The phylogenetic tree of culturable bacteria from six airborne samples. The red circle indicates the residential area **(A)**; the yellow square indicates the e-industrial park **(B)**; the purple triangle indicates the office area **(C)**; and the stars represent the waste transfer station **(D)**, when morning in green **(Dm)**, afternoon in brown **(Da)**, and night in gray **(Dn)**. The 16S rRNA sequences of all bacteria isolated from ambient air were aligned using the ClustalW program. The neighbor-joining tree was constructed using MEGA7.0. All branches have a bootstrapping value of at least 50% based on 500 bootstrap replicates. The pop-out table shows isolates with similarities of 100 or <98.7%.

A total of 229 strains were isolated from nutritional agar, 149 of which originated in e-waste disassembly sites. These bacteria belonged to 21 genera and 4 phyla (Figure 1). Firmicutes were the most heavily represented phylum in sites A (51.5%) and B (54.5%), followed by Actinobacteria (A: 39.4%, B: 27.3%). Conversely, Actinobacteria (49.0, 53.8, 72, and 78.6%, respectively) dominated at sites C, Dm, Da, and Dn, followed by Firmicutes (46.9, 42.9, 24.0, and 21.4%, respectively). Bacteroidetes were detected only at site C. At the genus level, *Bacillus* dominated sites A, B, C, and Dm, while *Microbium* and *Micrococcus* dominated in Da and Dn. The genus *Microbacterium* was represented at all sampling sites. Among the isolates were 17 strains of three species classified as conditional pathogenic bacteria, namely *Staphylococcus* (12/17), *Corynebacterium* (3/17), and *Pseudomonas* (2/17). These bacteria were mainly found in office area (7/17), followed by residential area (6/17), e-industrial park (2/17), and waste transfer station (2/17).

### Microbial Profiling Based on 16S rRNA Gene High-Throughput Sequencing

**Figure 2:**
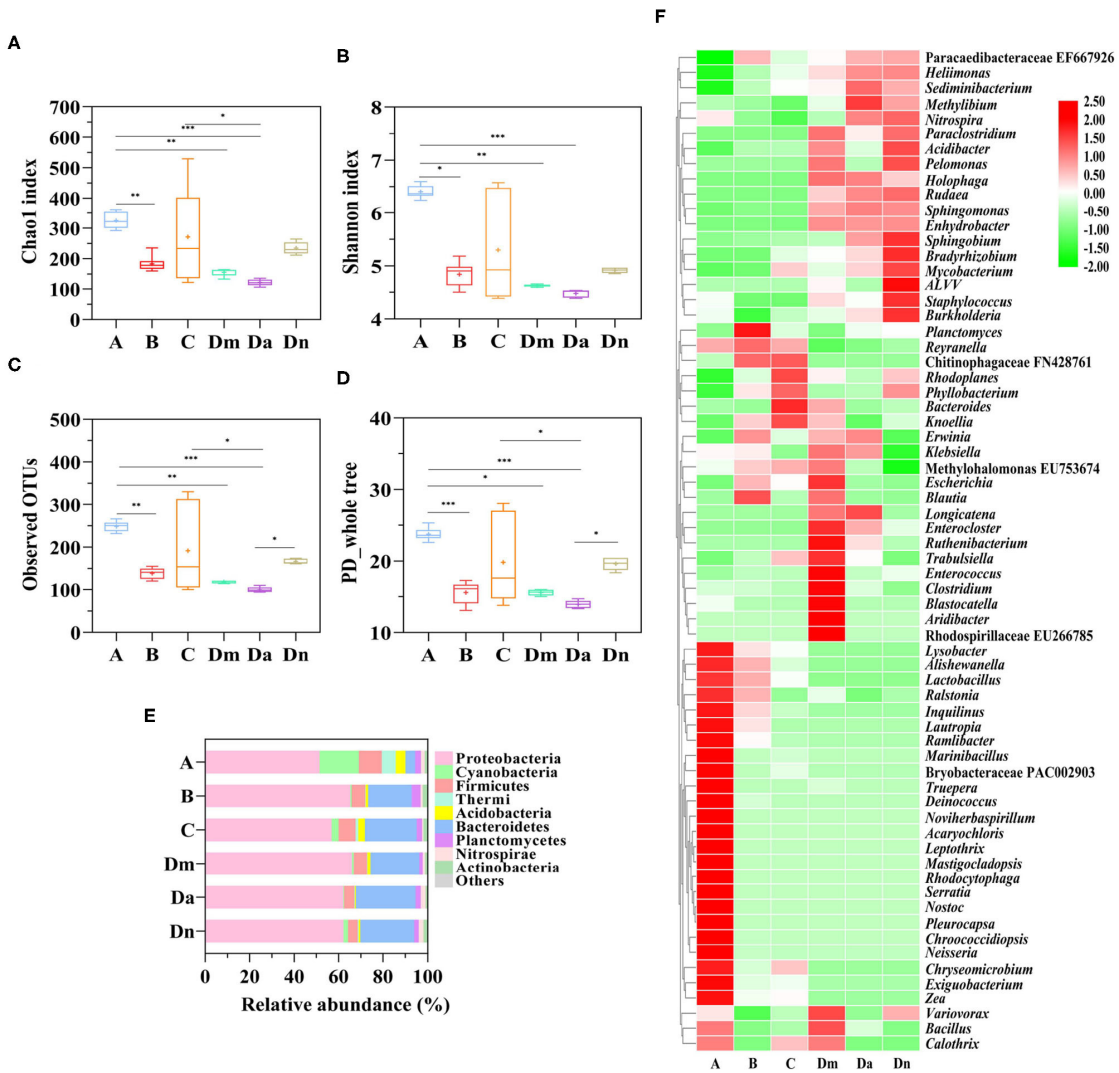
Alpha diversity of bacterial populations from six airborne samples. Boxplots of **(A)** the Chao1 index, **(B)** the Shannon index, **(C)** Observed OTUs, and **(D)** the PD whole tree of the ambient air samples. Differences were considered significant at the *P* < 0.05 (*), *P* < 0.01 (**), and *P* < 0.001 (***) levels. **(E)** The distributions of the predominant bacteria (the 9 most abundant within each group) at the phylum level. **(F)** Normalized heatmap of the identified phylotypes at the genus level (The top 70 bacteria in total abundance were shown), with high and low relative abundance indicated by red and green, respectively.

There were 1,529 bacterial Operational Taxonomic Units (OTUs) and 527,615 total counts acquired after high-throughput sequencing. The range of counts in the samples was 1,813–20,512, with a mean of 7,537 counts per sample. To evaluate the species richness and alpha diversity of the microbial communities, the number of observed OTUs and the Chao1 index, Shannon index, and PD whole tree index were calculated for each sample (Figures 2A–D). The alpha diversity index of airborne bacteria in site A was significantly higher than those for sites B, Dm, and Da (*P* < 0.05). Although Dn was higher than Da in the OTUs and PD whole tree index, when combined with the Chao1 and Shannon index, we believed that Da and Dn's alpha diversity was similar. The alpha diversity indices of e-industrial park, office area, and waste transfer station showed no statistical difference.

The community structures of the collected airborne bacteria differed between the sites. Proteobacteria (51.5–66.0%) was the most dominant phylum, followed by Bacteroidetes (4.3–26.9%), Firmicutes (4.4–10.4%), and Cyanobacteria (0.3–17.6%) (Figure 2E). Proteobacteria was mainly represented by Alpha-proteobacteria, Betaproteobacteria, and Gamma-proteobacteria. Alpha-proteobacteria had high relative abundances (58.86%). Bacteroidetes were mainly represented by Saprospirae and Cytophagia. Firmicutes were mainly represented by *Bacill, Clostridia*, and *Erysipelotrichi*. The relative abundance of Cyanobacteria and Thermi in the residential area was higher (*P* < 0.001) than in the other sites. Additionally, Bacteroidetes were more abundant in the electronic industrial park, office area, and waste transfer station than the residential area (*P* < 0.001).

**Figure 3:**
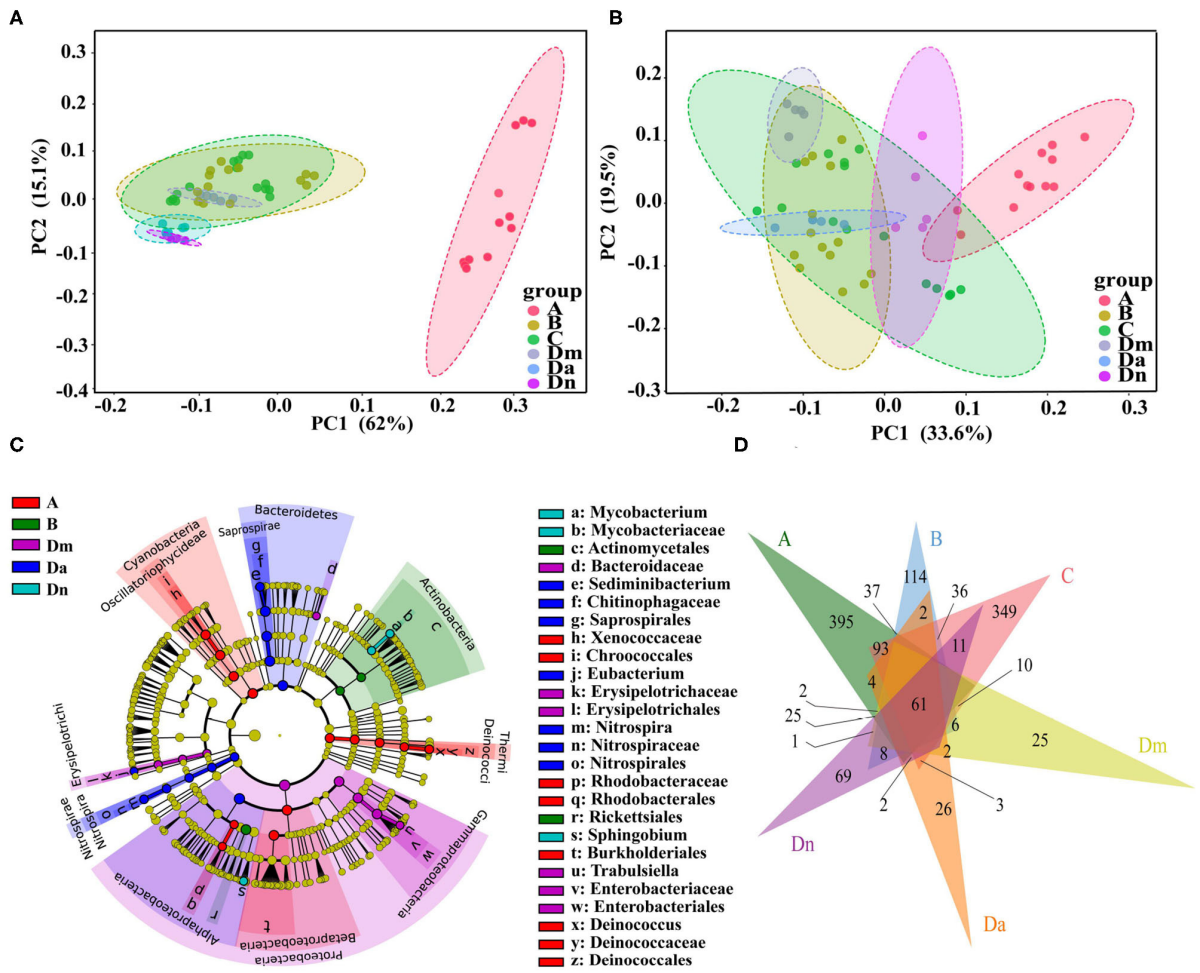
Beta diversity and taxonomic features of bacterial populations from six airborne samples. Principal coordinate analysis plots of **(A)** weighted and **(B)** unweighted UniFrac distances with samples colored by sampling site. The percentage of diversity captured by each coordinate is shown. **(C)** Cladogram for the taxonomic representation of significant differences among different sampling groups. The colored nodes from the inner to the outer circles represent taxa from the phylum to genus level. Significantly different taxa (linear discriminant analysis >3) are indicated by different colors representing the four groups (*P* < 0.05). **(D)** Venn diagram based on OTUs. Different colors represent different sampling sites. The overlaps represent the common taxa between sampling sites, and the non-overlapping portions represent unique taxa in each sampling site.

Principal coordinate analysis (PCoA) based on weighted UniFrac distance (beta diversity) with 62.0% explained variation for the x-axis and 15.1% for the y-axis (77.1% in total) (Figure 3A). The total diversity captured by the top two principal coordinates was 53.1% by unweighted UniFrac (Figure 3B). There were statistically significant differences in microbe composition between the sampling sites: the *R*^2^ values for the weighted and unweighted distances were 0.718 and 0.544, respectively (*P* < 0.001). LDA Effect Size (LEfSe) analysis showed that 6 bacterial genera were abundant in sites A, Dm, Da, and Dn (Figure 3C). As shown by the Venn diagram in Figure 3D, we identified 752, 395, 717, 191, 152, and 263 OTUs in samples A, B, C, Dm, Da, and Dn, respectively. Sixty-one OTUs were shared, comprising 3.99% of all identified OTUs. These results demonstrate the existence of differences in the structure and diversity in site-specific bacterial communities.

### Function Prediction by PICRUSt Analysis

Functional pathways associated with the identified bacterial strains communities were identified using their gene sequences and the Kyoto Encyclopedia of Genes and Genomes (KEGG), as shown in Supplementary Figure 3. The most abundant level 2 KEGG functional pathways across all samples were xenobiotics biodegradation and metabolism (6.1%), infectious diseases (5.2%), and lipid metabolism (5.2%). The most abundant level 3 function across all samples was Transporters, ABC transporters and DNA repair and recombination proteins. It should also be noted that membrane transport genes were identified in all samples, indicating that energy metabolism was important to the studied bacterial communities (23). Analysis of the predicted aerosol bacterial community functions for samples from six sites revealed no differences in the copy number sequences of predicted genes in tertiary functional layers. Interestingly, the six samples exhibited no differences in predicted function structure despite clear differences in bacterial species composition. This is probably because airborne bacterial communities require certain common functions. However, further study will be needed to identify these functions and their relevance to these communities.

## Discussion

The airborne bacteria at the six sampling points differed in concentration and particle size distribution. These eigenvalues varied in relation to the environmental characteristics of the sampling sites, sampling times, and human activities. Residential area (A), office area (C), and waste transfer station in afternoon (Da) had high concentrations of culturable airborne bacteria. The bacterial concentrations in the C and Da aerosols exceeded 700 CFU/m^3^, which is the threshold above which airborne bacteria may significantly affect the occurrence of airborne infection according to Parker (24). The characteristics of these environments may be the main cause of the high levels of bacteria. High concentrations of cultivable bacteria have previously been detected in plant-rich and densely populated sites such as offices (20, 25), which is unsurprising because both leaves and human skin could greatly increase numbers of indoor bacteria (26, 27). Also, sampling in the office area occurred during off-hours, when temperatures were high and there was frequent movement of people. Such high levels of human activity could have re-suspended precipitated bacteria into the air. Likewise, the complex demographic composition of the residential area (A) contributed to the high bacterial concentrations.

In this study, the bacterial concentrations did not correlate with temperature or relative humidity (Supplementary Table 2) even though meteorological conditions can influence bioaerosols (28). It was possible that other influences were playing a decisive role. Sampling time and human activities also shaped the characteristics of airborne bacteria, such as, waste transfer station. The high bacterial concentrations in the afternoon at waste transfer station were also related to human activity. The particle distributions at the different time periods varied appreciably. Many factors may explain this outcome, such as differences in temperature and bacterial sources (28, 29). The impact of human activities was observed at the waste transfer station in the afternoon, when large amounts of floating bioaerosols were produced during sorting and transporting of accumulated waste (Da). Lower bacterial concentrations were observed in the morning, when sorting and transfer were not performed (Dm). The dominant bacterial particle size at the waste disposal site in the afternoon was also larger than that in the morning (when sorting and transfer were not done), in keeping with the results of an earlier study conducted in Poland (13). The particle size distribution at night (Dn) was dominated by coarse particles because the residual garbage was cleaned with a water gun in the evening, causing large amounts of coarse particulate matter to spatter (30).

Both culture-dependent and culture-independent methods found different airborne bacterial communities at different sampling points, with differences not only in diversity indices, but also in the abundance of bacteria at different levels (e.g., phylum or genus). For the cultivation method, a similar study identified Firmicutes and Actinobacteria as the most abundant bacterial phyla in aerosols (31). However, at the genus level, the dominant bacteria varied widely between sites, with the exception of *Bacillus* (32–34). High concentrations of *Bacillus* have been suggested to cause local infections (35); this was attributed to its ability to produce elliptical endospores that enable adaptation to harsh environmental conditions *via* extended periods of dormancy (34).

Of the 229 strains that were isolated above, 14 were found to be potentially novel strains, with a 16S rRNA gene sequence similarity below 98.65% (Figure 1), a threshold value used to differentiate species (36). These bacteria were classified as *Agrococcus, Bacillus, Brachybacterium, Herbaspirillum, Janibacter, Lysinibacillus, Microbacterium, Nocardioides, Porphyrobacter, Pseudarthrobacter, Psychrobacter, Quadrisphaera, Roseicella, and Yonghaparkia* at the genus level, especially *Roseicella sp*. GB24, a novel bacterial genus previously isolated from an air conditioning system in South Korea (37). A detailed list could be found in Supplementary Table 3. Most of them had not previously been detected or were only rarely detected in the atmosphere. *Bacillus*, as mentioned above, can adapt to a variety of environments. *Microbacterium* produces a pigment that protects other microorganisms from radiation (38). Their ability to resist or tolerate specific adverse environmental factors and stand out from their crowd under the selective pressure of waste may partly explain why these bacteria could become potential novel species and isolated in waste-related sites.

Of these 14 potentially novel strains, 9 (2 from the office area, 4 from the residential area, 3 from the e-industrial park) were collected in the e-waste dismantling site, and 5 from the waste transfer station. The proportion of airborne fine particulate matter (PM) decreased in the e-waste environment (39). The reduction of fine PM with a large surface area prevented nutrients from being well-enriched. The oligotrophic state drove changes in bacteria, leading to the possibility of the evolution of novel bacteria (40, 41). The sequencing results showed that at all sampling points, the proportion of uncultivated bacteria in the e-waste sites was highest for the dominant genus. These 14 strains have not previously been detected in the atmosphere, indicating that the e-waste area and waste transfer station therefore may have unique bacterial diversity.

Unlike culturable bacterial concentrations, the residential area had the highest alpha diversity, which may be related to its characteristics. Residential areas were complex environments with variable population compositions; consequently, they can vary widely in terms of temperature, humidity, availability of air conditioning, pets, the presence of infants and young children, and level of urbanization, all of which could affect bacterial diversity; high alpha diversity may be a manifestation of this variability (42, 43). The airborne coarse PM (2.5–10 μm) in e-waste was 3–4 times more abundant than fine/ultrafine PM (<2.5 μm) (39). High concentrations of heavy metal ions, such as Ni, Pb, and Zn, were attached to these coarse PM. The processing of e-waste also released high concentrations of organic pollutants, such as polybrominated diphenyl ethers, into the air (44). High exposure to heavy metals and organic pollutants both contributed to the reduction of bacterial diversity in e-industrial park (45, 46). Given the likely toxicity of e-waste pollutants toward bacteria, it was unsurprising that the electronic industrial park aerosol had the lowest levels of microbial diversity (47, 48).

For the non-culture method, different bacterial community structures have been observed in other regions; for example, Proteobacteria comprised 50–80% of the bacterial community in PM_2.5_ from Beijing and Shanghai, while Firmicutes was the most abundant phylum in Guilin, followed by Proteobacteria (34). In general, Proteobacteria and Firmicutes seem to be the most abundant airborne phyla, but there appears to be site-specific variation in their relative abundance (49). Genus-level distribution data are presented in Figure 2F. Paracaedibacteraceae uncultured bacteria EF667926 and Chitinophagaceae uncultured bacteria FN428761 comprised over 30% of the total genus-level bacterial population in sites B and C. A high proportion of the detected strains were unknown or poorly understood (uncultured) (50) suggesting that the waste-associated aerosols were quite different from those in other places, which may be rich repositories of previously uncultured bacteria.

High-throughput sequencing showed that Proteobacteria was the most abundant phylum, followed by Bacteroidetes. However, only a few Proteobacteria strains and one Bacteroidetes strain could be isolated. This may be because airborne bacteria have limited ability to survive in a state amenable to culture after collection and atomization in a sampler (51). Conversely, *Bacillus* was the most frequently isolated bacterial genus in nutritional agar, but was outside the top 10 bacterial genera by abundance based on the high-throughput sequencing results. Most bacterial strains identified by high-throughput sequencing were Gram-negative (55.8–89.0%), but 91.7% of those isolated by culture were Gram-positive. This may be related to the loss of cultivability of Gram-negative bacteria after atomization (51). High levels of Gram-negative bacteria can indirectly lead to an increase in endotoxins in the air (52). Additionally, cultivation was performed on nutritional agar, which may be unsuitable for certain bacteria. Finally, cells out of the cell division cycle would also not be cultivable (53). Combining next-generation sequencing methods with a culture-dependent method thus increases the diversity of microorganisms that can be detected.

This work investigated the airborne bacteria of waste-related areas with culture-dependent and culture-independent methods. Based on both culture and sequencing data, we concluded that bacterial aerosols in e-waste associated environment and waste transfer station exhibited different and unique diversity. From the perspective of the culture method, the cultivable airborne bacteria in these two places had different concentrations, particle sizes and abundances. Human activities may partly explain why this difference occurs. Fourteen potentially novel strains of bacteria were isolated in the waste-related environment, mainly distributed in the e-waste dismantling site. In terms of sequencing results, differences in genus levels and 3.99% of the shared OTUs indicated differences in air bacteria between the two places, despite the differences of diversity indices only existed in the residential area. However, practical constraints prevented us from sampling in non-e-waste or non-waste environments near the e-waste dismantling sites, and the limited number of samples weakens the strength of our evidence. Nevertheless, we still believe that aerosols in the waste associated environment have unique bacterial diversity.

## Data Availability Statement

The datasets presented in this study can be found in online repositories. The names of the repository/repositories and accession number(s) can be found below: https://www.ncbi.nlm.nih.gov/, MT214102–MT214329, MT218357, and MT214107; https://www.ncbi.nlm.nih.gov/, PRJEB38065.

## Author Contributions

GZ: conceptualization, methodology, supervision, and writing - review and editing. YP: investigation and writing - original draft. QR: resources. PC: formal analysis. JW: validation. ZW: formal analysis. All authors contributed to the article and approved the submitted version.

## Conflict of Interest

The authors declare that the research was conducted in the absence of any commercial or financial relationships that could be construed as a potential conflict of interest.

## References

[1] Cao C Jiang WJ Wang BY Fang JH Lang JD Tian G. Inhalable microorganisms in Beijing's PM_2.5_ and PM_10_ pollutants during a severe smog event. Environ Sci Technol. (2014) 48:1499–507. 10.1021/es4048472 24456276 PMC3963435

[2] Estaki M Pither J Baumeister P Little JP Gill SK Ghosh S. Cardiorespiratory fitness as a predictor of intestinal microbial diversity and distinct metagenomic functions. Microbiome. (2016) 4:42. 10.1186/s40168-016-0189-7 27502158 PMC4976518

[3] Peleg AY Hogan DA Mylonakis E. Medically important bacterial-fungal interactions. Nat Rev Microbiol. (2010) 8:340–9. 10.1038/nrmicro2313 20348933

[4] Orland C Emilson E Basiliko N Mykytczuk N Gunn JM Tanentzap AJ. Microbiome functioning depends on individual and interactive effects of the environment and community structure. ISME J. (2019) 13:1–11. 10.1038/s41396-018-0230-x 30042502 PMC6298968

[5] Chen HW Nwe PK Yang Y Rosen CE Bielecka AA Kuchroo M. A forward chemical genetic screen reveals gut microbiota metabolites that modulate host physiology. Cell. (2019) 177:1217–31. 10.1016/j.cell.2019.03.036 31006530 PMC6536006

[6] Long-Smith C O'Riordan KJ Clarke G Stanton C Dinan TG Cryan JF. Microbiota-gut-brain axis: new therapeutic opportunities. Annu Rev Pharmacol. (2020) 60:477–502. 10.1146/annurev-pharmtox-010919-023628 31506009

[7] Overmann J Abt B Sikorski J. Present and future of culturing bacteria. Annu Rev Microbiol. (2017) 71:711–30. 10.1146/annurev-micro-090816-093449 28731846

[8] Alsved M Fraenkel CJ Bohgard M Widell A Söderlund-Strand A Lanbeck P. Sources of airborne norovirus in hospital outbreaks. Clin Infect Dis. (2020) 70:2023–8. 10.1093/cid/ciz584 31257413 PMC7201413

[9] Mirskaya E Agranovski IE. Sources and mechanisms of bioaerosol generation in occupational environments. Crit Rev Microbiol. (2018) 44:739–58. 10.1080/1040841X.2018.1508125 30318982

[10] Chen YH Yan C Yang YF Ma JX. Quantitative microbial risk assessment and sensitivity analysis for workers exposed to pathogenic bacterial bioaerosols under various aeration modes in two wastewater treatment plants. Sci Total Environ. (2020) 755:142615. 10.1016/j.scitotenv.2020.142615 33038813 PMC7527313

[11] Krystosik A Njoroge G Odhiambo L Forsyth JE Mutuku F LaBeaud AD. Solid wastes provide breeding sites, burrows, and food for biological disease vectors, and urban zoonotic reservoirs: a call to action for solutions-based research. Front Public Health. (2020) 7:405. 10.3389/fpubh.2019.00405 32010659 PMC6979070

[12] Schwarzer S De Bono A Giuliani G Kluser S Peduzzi P. E-waste, the hidden side of IT equipment's manufacturing and use. United Nations Environment Programme (2005). Retrieved from: https://archive-ouverte.unige.ch/unige:23132

[13] Cyprowski M Ławniczek-Wałczyk A Gołofit-Szymczak M Fraczek K Kozdrój J Górny RL. Bacterial aerosols in a municipal landfill environment. Sci Total Environ. (2019) 660:288–96. 10.1016/j.scitotenv.2018.12.356 30640097

[14] Ghoul M Mitri S. The ecology and evolution of microbial competition. Trends Microbiol. (2016) 24:833–45. 10.1016/j.tim.2016.06.011 27546832

[15] Madsen AM Frederiksen MW Bjerregaard M Tendal K. Measures to reduce the exposure of waste collection workers to handborne and airborne microorganisms and inflammogenic dust. Waste Manag. (2020) 101:241–9. 10.1016/j.wasman.2019.10.023 31630069

[16] Blainey PC. The future is now: single-cell genomics of bacteria and archaea. FEMS Microbiol Rev. (2013) 37:407–27. 10.1111/1574-6976.12015 23298390 PMC3878092

[17] Nguyen TM Seo C Ji M Paik MJ Myung SW Kim J. Effective soil extraction method for cultivating previously uncultured soil bacteria. Appl Environ Microbiol. (2018) 84:e01145–18. 10.1128/AEM.01145-18 30291118 PMC6275337

[18] Pham VH Kim J. Cultivation of unculturable soil bacteria. Trends Biotechnol. (2012) 30:475–84. 10.1016/j.tibtech.2012.05.007 22770837

[19] Schloss PD Girard RA Martin T Edwards J Thrash JC. Status of the archaeal and bacterial census: an update. mBio. (2016) 7:e00201–16. 10.1128/mBio.00201-16 27190214 PMC4895100

[20] Li XY Chen HX Yao MS. Microbial emission levels and diversities from different land use types. Environ Int. (2020) 143:105988. 10.1016/j.envint.2020.105988 32717647

[21] Wagner AO Praeg N Reitschuler C Illmer P. Effect of DNA extraction procedure, repeated extraction and ethidium monoazide (EMA)/propidium monoazide (PMA) treatment on overall DNA yield and impact on microbial fingerprints for bacteria, fungi and archaea in a reference soil. Appl Soil Ecol. (2015) 93:56–64. 10.1016/j.apsoil.2015.04.005 26339125 PMC4461152

[22] He Y Wu W Wu S Zheng HM Li P Sheng HF. Linking gut microbiota, metabolic syndrome and economic status based on a population-level analysis. Microbiome. (2018) 6:172. 10.1186/s40168-018-0557-6 30249275 PMC6154942

[23] Brothers CJ Van Der Pol WJ Morrow CD Hakim JA Koo H McClintock JB. Ocean warming alters predicted microbiome functionality in a common sea urchin. Proc Biol Sci. (2018) 285:20180340. 10.1098/rspb.2018.0340 29925614 PMC6030520

[24] Parker MT. Hospital-Acquired Infections: Guidelines to Laboratory Methods. Copenhagen: World Health Organization. Regional Office for Europe (1978).

[25] Mukherjee N Dowd SE Wise A Kedia S Vohra V Banerjee P. Diversity of bacterial communities of fitness center surfaces in a U.S. metropolitan area. Int J Environ Res Public Health. (2014) 11:12544–61. 10.3390/ijerph111212544 25479039 PMC4276630

[26] Mhuireach G Johnson BR Altrichter AE Ladau J Meadow JF Pollard KS. Urban greenness influences airborne bacterial community composition. Sci Total Environ. (2016) 571:680–7. 10.1016/j.scitotenv.2016.07.037 27418518

[27] Timm CM Loomis K Stone W Mehoke T Brensinger B Pellicore M. Isolation and characterization of diverse microbial representatives from the human skin microbiome. Microbiome. (2020) 8:58. 10.1186/s40168-020-00831-y 32321582 PMC7178971

[28] Zhai YB Li X Wang TF Wang B Li CT Zeng GM. A review on airborne microorganisms in particulate matters: Composition, characteristics and influence factors. Environ Int. (2018) 113:74–90. 10.1016/j.envint.2018.01.007 29421410

[29] Romano S Di Salvo M Rispoli G Alifano P Perrone MR Talà A. Airborne bacteria in the central mediterranean: Structure and role of meteorology and air mass transport. Sci Total Environ. (2019) 697:134020. 10.1016/j.scitotenv.2019.134020 31491629

[30] Prussin AN Marr LC. Sources of airborne microorganisms in the built environment. Microbiome. (2015) 3:78. 10.1186/s40168-015-0144-z 26694197 PMC4688924

[31] Lee SH Lee HJ Kim SJ Lee HM Kang H Kim YP. Identification of airborne bacterial and fungal community structures in an urban area by T-RFLP analysis and quantitative real-time PCR. Sci Total Environ. (2010) 408:1349–57. 10.1016/j.scitotenv.2009.10.061 19913878

[32] Li H Zhou XY Yang XR Zhu YG Hong YW Su JQ. Spatial and seasonal variation of the airborne microbiome in a rapidly developing city of China. Sci Total Environ. (2019) 665:61–8. 10.1016/j.scitotenv.2019.01.367 30772579

[33] Xu CH Wei M Chen JM Wang XF Zhu C Li JR. Bacterial characterization in ambient submicron particles during severe haze episodes at Ji'nan, China. Sci Total Environ. (2017) 580:188–96. 10.1016/j.scitotenv.2016.11.145 28017418

[34] Zhong S Zhang LS Jiang XY Gao P. Comparison of chemical composition and airborne bacterial community structure in PM_2.5_ during haze and non-haze days in the winter in Guilin, China. Sci Total Environ. (2019) 655:202–10. 10.1016/j.scitotenv.2018.11.268 30471588

[35] Pahari AK Dasgupta D Patil RS Mukherji S. Emission of bacterial bioaerosols from a composting facility in Maharashtra, India. Waste Manag. (2016) 53:22–31. 10.1016/j.wasman.2016.04.027 27155946

[36] Kim M Oh HS Park SC Chun J. Towards a taxonomic coherence between average nucleotide identity and 16S rRNA gene sequence similarity for species demarcation of prokaryotes. Int J Syst Evol Microbiol. (2014) 64:346–51. 10.1099/ijs.0.059774-0 24505072

[37] Khan SA Jeong SE Jung HS Quan ZX Jeon CO. *Roseicella frigidaeris* gen. nov., sp. nov., isolated from an air-conditioning system. Int J Syst Evol Microbiol. (2019) 69:1384–9. 10.1099/ijsem.0.003322 30816841

[38] Fang ZG Yao WC Lou XQ Hao CM Gong CJ Ouyang ZY. Profile and characteristics of culturable airborne bacteria in Hangzhou, southeast of China. Aerosol Air Qual Res. (2016) 16:1690–700. 10.4209/aaqr.2014.11.0274

[39] Kim YH Wyrzykowska-Ceradini B Touati A Krantz QT Dye JA Linak WP. Characterization of size-fractionated airborne particles inside an electronic waste recycling facility and acute toxicity testing in mice. Environ Sci Technol. (2015) 49:11543–50. 10.1021/acs.est.5b03263 26332991

[40] Brewer TE Aronson EL Arogyaswamy K Billings SA Botthoff JK Campbell AN. Ecological and genomic attributes of novel bacterial taxa that thrive in subsurface soil horizons. mBio. (2019) 10:e01318–19. 10.1101/647651 31575762 PMC6775450

[41] Props R Monsieurs P Vandamme P Leys N Denef VJ Boon N. Gene expansion and positive selection as bacterial adaptations to oligotrophic conditions. mSphere. (2019) 4:e00011–9. 10.1128/mSphereDirect.00011-19 30728279 PMC6365617

[42] Dannemiller KC Gent JF Leaderer BP Peccia J. Influence of housing characteristics on bacterial and fungal communities in homes of asthmatic children. Indoor Air. (2016) 26:179–92. 10.1111/ina.12205 25833176 PMC4591094

[43] Mccall L Callewaert C Zhu Q Song SJ Bouslimani A Minich JJ. Home chemical and microbial transitions across urbanization. Nat Microbiol. (2020) 5:108–15. 10.1038/s41564-019-0593-4 31686026 PMC7895447

[44] Li TY Zhou JF Wu CC Bao LJ Shi L Zeng EY. Characteristics of polybrominated diphenyl ethers released from thermal treatment and open burning of e-waste. Environ Sci Technol. (2018) 52:4650–7. 10.1021/acs.est.8b00780 29600707

[45] Wu ZN Gao GH Wang YY. Effects of soil properties, heavy metals, and PBDEs on microbial community of e-waste contaminated soil. Ecotox Environ Saf. (2019) 180:705–14. 10.1016/j.ecoenv.2019.05.027 31151067

[46] Wu QH Du YM Huang ZY Gu JD Leung JYS Mai BX. Vertical profile of soil/sediment pollution and microbial community change by e-waste recycling operation. Sci Total Environ. (2019) 669:1001–10. 10.1016/j.scitotenv.2019.03.178 30970449

[47] Dvorák P Nikel PI Damborský J de Lorenzo V. Bioremediation 3.0: Engineering pollutant-removing bacteria in the times of systemic biology. Biotechnol Adv. (2017) 35:845–66. 10.1016/j.biotechadv.2017.08.001 28789939

[48] Jiang LF Luo CL Zhang DY Song MK Sun YT Zhang G. Biphenyl-metabolizing microbial community and a functional operon revealed in e-waste-contaminated soil. Environ Sci Technol. (2018) 52:8558–67. 10.1021/acs.est.7b06647 29733586

[49] Park EH Heo J Kim H Yi SM. The major chemical constituents of PM_2.5_ and airborne bacterial community phyla in Beijing, Seoul, and Nagasaki. Chemosphere. (2020) 254:126870. 10.1016/j.chemosphere.2020.126870 32353811

[50] Cao Y Yu XW Ju F Zhan HC Jiang B Kang H. Airborne bacterial community diversity, source and function along the Antarctic Coast. Sci Total Environ. (2020) 765:142700. 10.1016/j.scitotenv.2020.142700 33069481

[51] Peccia J Hernandez M. Incorporating polymerase chain reaction-based identification, population characterization, and quantification of microorganisms into aerosol science: a review. Atmos Environ. (2006) 40:3941–61. 10.1016/j.atmosenv.2006.02.029 32288550 PMC7108281

[52] Wang C Zhang ZW Liu H. Microwave-induced release and degradation of airborne endotoxins from *Escherichia coli* bioaerosol. J Hazard Mater. (2019) 366:27–33. 10.1016/j.jhazmat.2018.11.088 30500695 PMC7116933

[53] Simu K Holmfeldt K Zweifel UL Hagström A. Culturability and coexistence of colony-forming and single-cell marine bacterioplankton. Appl Environ Microbiol. (2005) 71:4793–800. 10.1128/AEM.71.8.4793-4800.2005 16085877 PMC1183315

