# Supplementary material for: Insight Into Microbial Community Aerosols Associated With Electronic Waste Handling Facilities by Culture-Dependent and Culture-Independent Methods
Source: Front Public Health. 2021 Apr 6;9:657784. doi: 10.3389/fpubh.2021.657784 (PMC8055949; doi:10.3389/fpubh.2021.657784)
Supplement: Supplementary file 1 [file Data_Sheet_1.docx]

**Supplementary Material**

**Supplementary Table S1** The concentrations of culturable bacterial aerosols and weather parameters

| Sampling site | Concentration  (CFU/m^3)^ | Temperature  (°C) | Humidity  (%) | Wind direction | Wind speed  (m/s) | time |
| --- | --- | --- | --- | --- | --- | --- |
| A (residential area) | 438 ± 180 | 23 | 74 | Northeast | 5.6 | 9:00 |
| B (e-industrial park) | 70 ± 5 | 28 | 54 | Northeast | 5.6 | 13:00 |
| C (office area) | 744 ± 78 | 29 | 45 | Northeast | 5.6 | 17:00 |
| Dm (waste transfer station) | 124 | 23 | 57 | Northeast | 0.9 | 10:00 |
| Da (waste transfer station) | 749 | 25 | 30 | Northeast | 0.9 | 16:00 |
| Dn (waste transfer station) | 516 | 21 | 47 | Northeast | 0.9 | 20:00 |

**Supplementary Table S2** Correlations between the bacterial concentrations and factors of temperature or humidity

|  | Sampling site | Temperature | | | Humidity | | |
| --- | --- | --- | --- | --- | --- | --- | --- |
|  |  | *r* | | *P*-value | *r* | | *P*-value |
| Concentration | Guiyu | 0.934 | 0.103 | | -0.252 | 0.838 | |
|  | Waste transfer station | 0.369 | 0.760 | | 0.077 | 0.951 | |

Pearson correlation coefficient and *P* < 0.05 were statistically significant.

**Supplementary Table S3** List of 14 potentially novel strains

| ID | | Speciesc | 16S rRNA gene sequence similarity (%) | Sampling site |
| --- | --- | --- | --- | --- |
| G223  GA224  GA68  NA57  GB41  GB23  GB24  GC16  NC21  MF12  MF31  MF43  NL221  NL45 | *Pseudarthrobacter oxydans*  *Yonghaparkia alkaliphila*  *Porphyrobacter sanguineus*  *Bacillus vireti*  *Herbaspirillum denitrificans*  *Nocardioides litoris*  *Roseicella uncultured*  *Janibacter limosus*  *Quadrisphaera granulorum*  *Lysinibacillus odysseyi*  *Psychrobacter ciconiae*  *Microbacterium thalassium*  *Agrococcus terreus*  *Brachybacterium sacelli* | | 98.50  98.60  96.00  98.58  97.86  98.23  97.79  98.30  98.28  97.00  96.88  98.60  98.60  96.61 | A  A  A  A  B  B  B  C  C  Da  Da  Da  Dn  Dn |


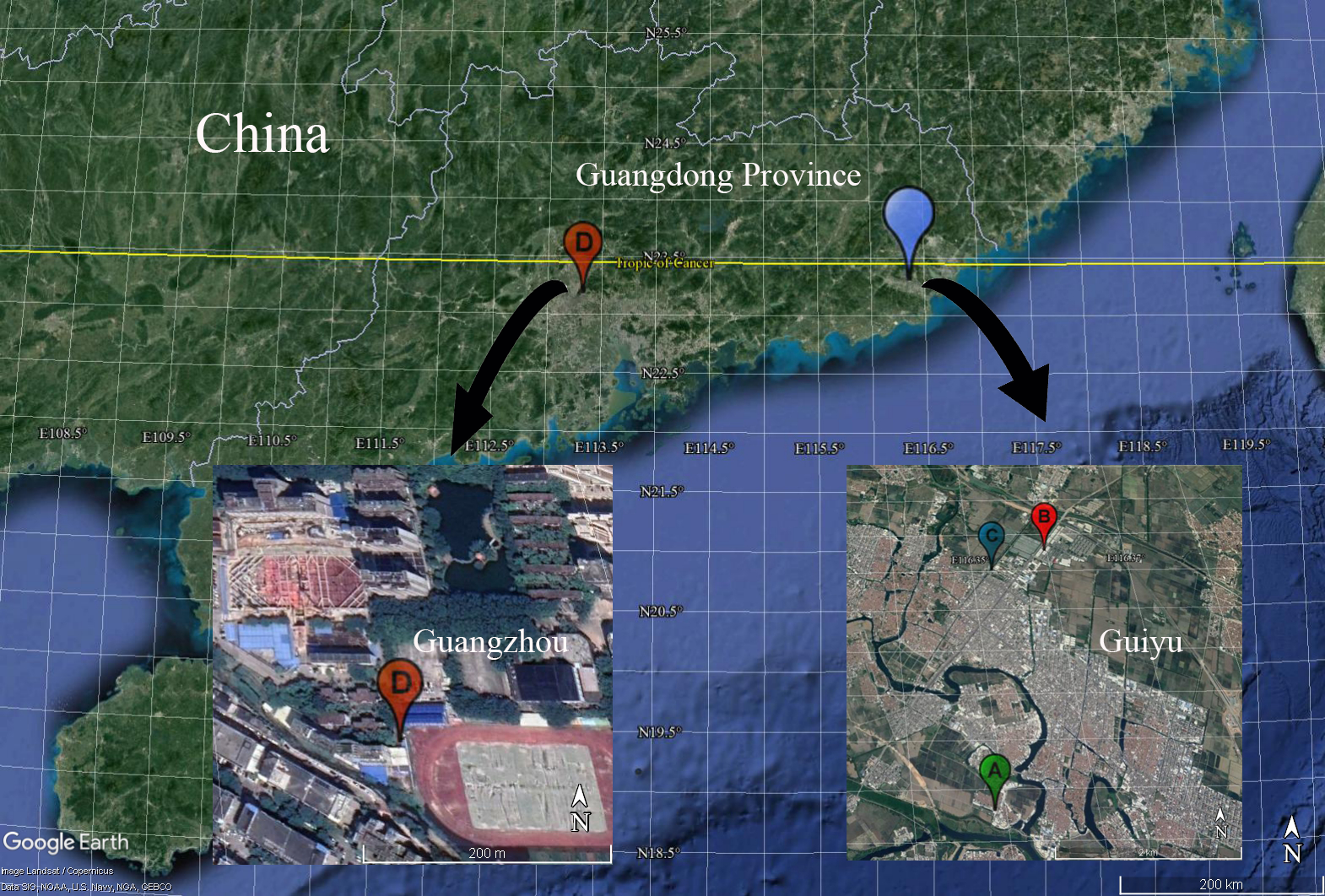


**Supplementary Figure. S1** Distribution map of sampling sites. The sampling sites included a residential area (A), an electronic industrial park (B), an office area (C), and a waste transfer station (D).


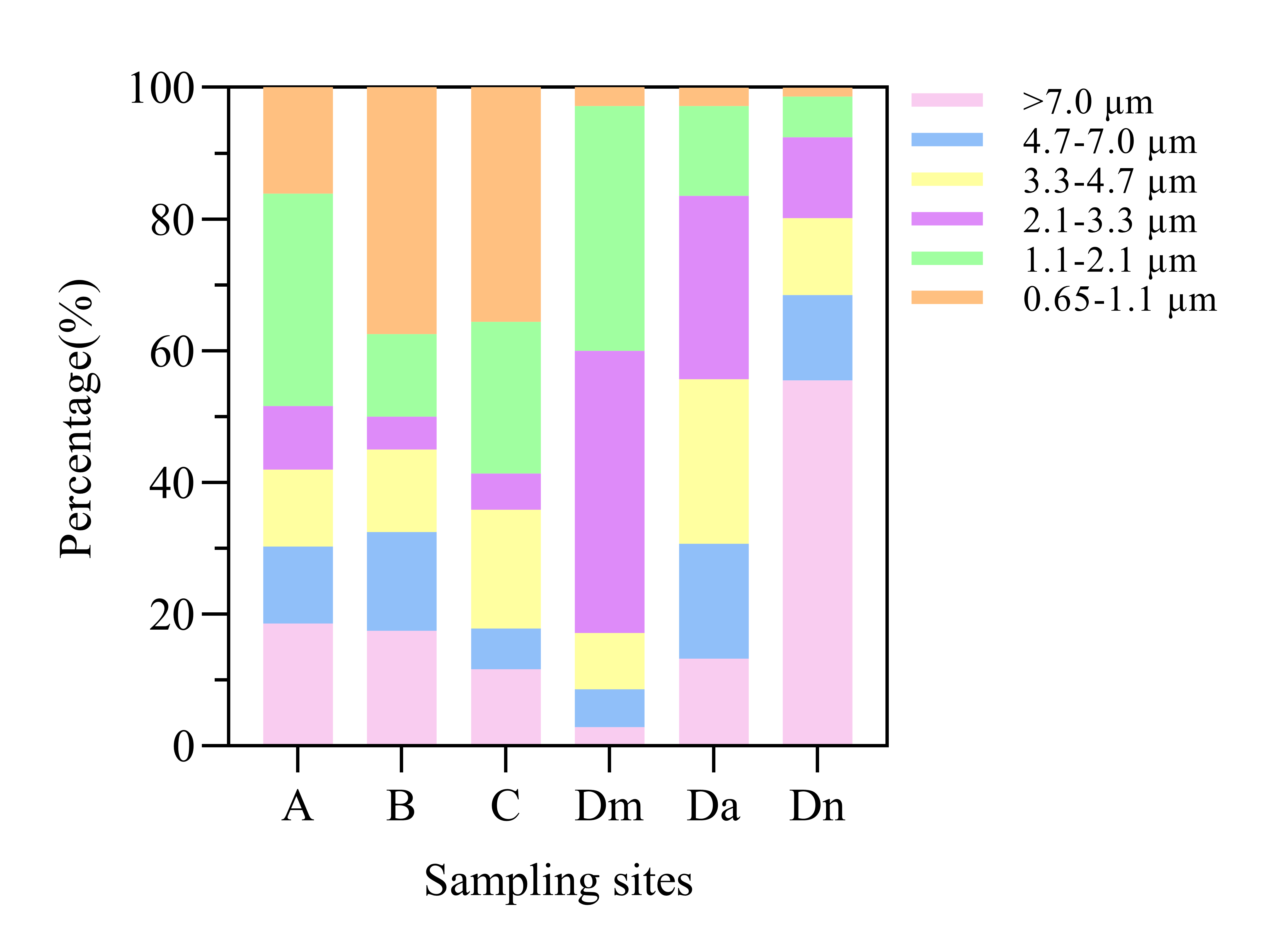


**Supplementary Figure. S2** Culturable bacterial particle size distribution histogram. A, B, C, Dm, Da, and Dn were indicates a residential area, electronic industrial park, office area and a waste transfer station in the morning, afternoon, and evening.


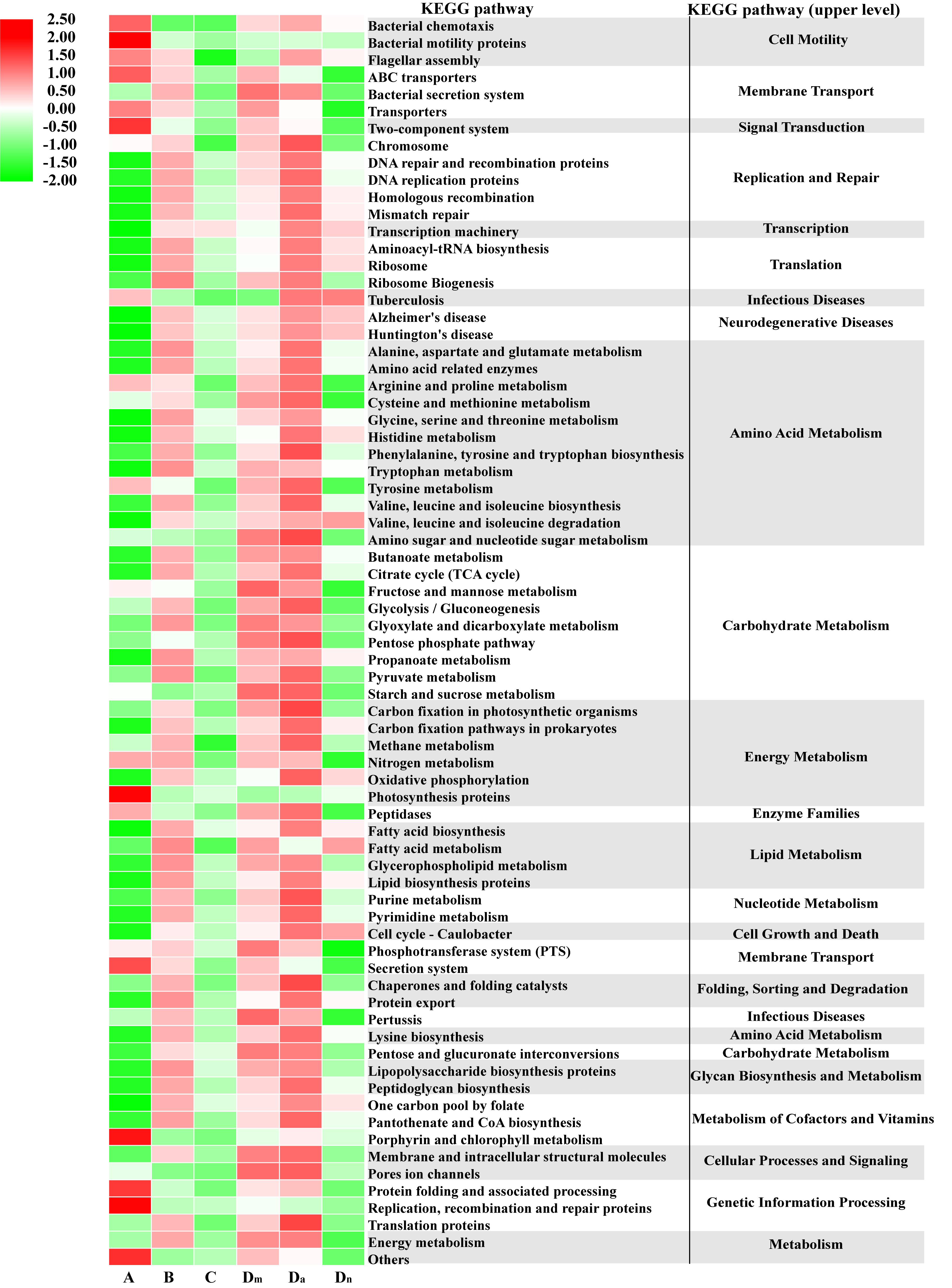


**Supplementary Figure. S3** Functional prediction of bacterial metagenome. To predict the metagenome function, heatmap of PICRUSt analysis showed significant KEGG pathway between level 2 and level 3.
